# miR-6883 downregulates HIF1α in colorectal and breast cancer cells

**DOI:** 10.17912/micropub.biology.000978

**Published:** 2024-01-26

**Authors:** Nicole A. Jensen-Velez, Lindsey Carlsen, Wafik S. El-Deiry

**Affiliations:** 1 Laboratory of Translational Oncology and Experimental Cancer Therapeutics, Department of Pathology and Laboratory Medicine, The Warren Alpert Medical School, Brown University, Providence, RI 02903, USA; 2 University of Puerto Rico, Arecibo PR 00612, USA; 3 Brown University, Providence, Rhode Island, United States; 4 Graduate Program in Pathobiology, The Warren Alpert Medical School, Brown University, Providence, RI 02903, USA; 5 Joint Program in Cancer Biology, Brown University and The Lifespan Health System, Providence, RI 02903, USA; 6 Legorreta Cancer Center at Brown University, The Warren Alpert Medical School, Brown University, Providence, RI 02903, USA; 7 Hematology-Oncology Division, Brown University and The Lifespan Cancer Institute, Providence, RI 02903, USA.

## Abstract

Colorectal cancer (CRC) and breast cancer (BC) are deadly diseases that rank as the second and fourth leading causes of cancer-related deaths, respectively. We have previously shown that miR-6883 targets CDK4/6 and that palbociclib-mediated CDK4/6 inhibition destabilizes HIF1α. We hypothesize that miR-6883 downregulates HIF1α in CRC and BC cells. miR-6883 was transfected into cells under normoxia or hypoxia and western blot analysis revealed that miR-6883 downregulates CDK4/6 and HIF1α in CRC and BC cells, pointing to miR-6883 as a promising therapeutic to target hypoxic tumors or HIF1α-deregulated cancer cells. Future studies will further investigate miR-6883 as a cancer biomarker, effects on HIF–related proteins, and therapeutic uses *in vivo* .

**Figure 1. miR-6883 downregulates CDK4/6 and HIF1α in colorectal and breast cancer cells:**
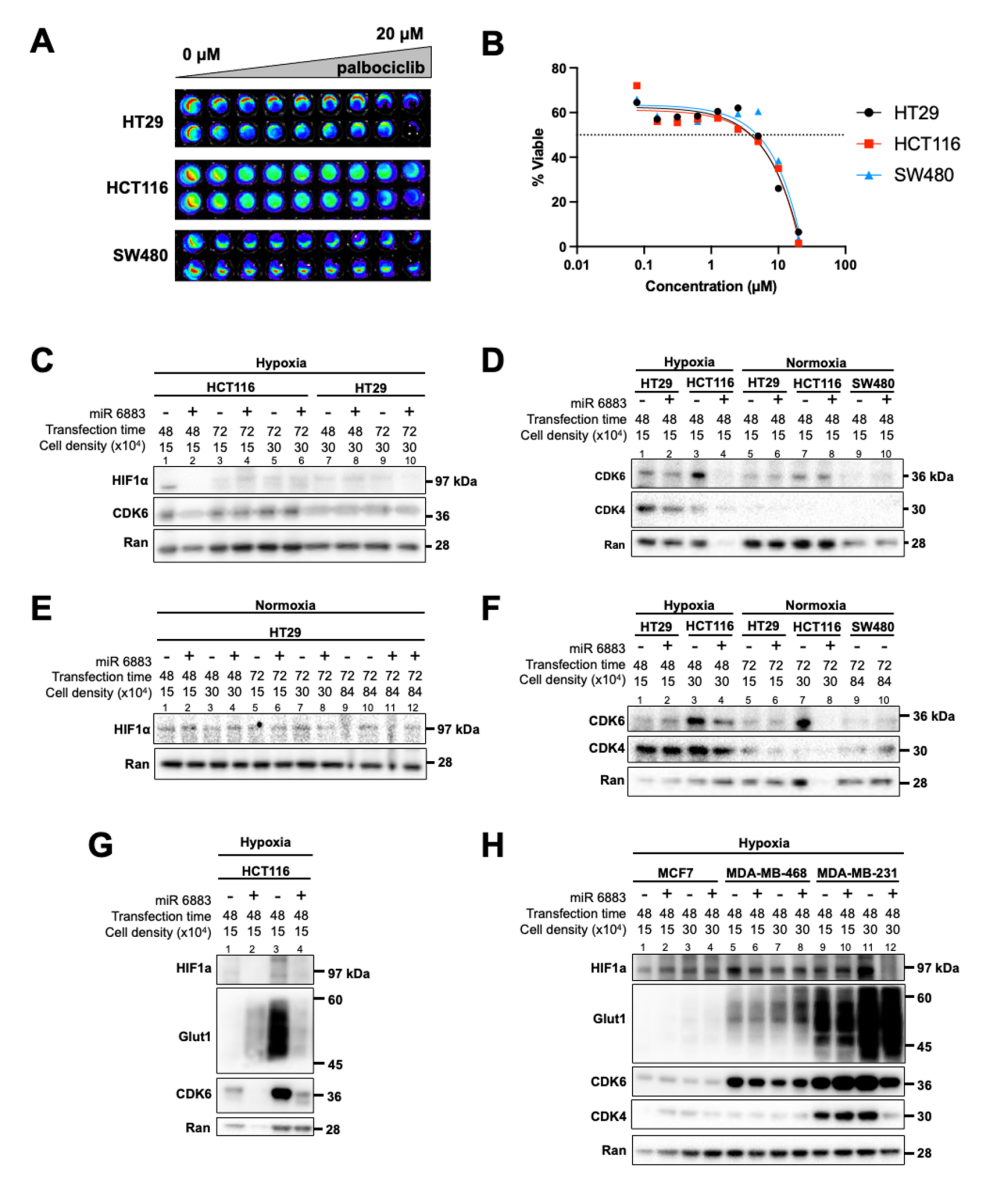
A) Colorectal cancer cells were treated with doses of palbociclib ranging from 0-20 µM. Cell viability was measured by imaging the bioluminescent signal after addition of CellTiter-Glo reagent. B) Percent viability of colorectal cancer cells treated with palbociclb was calculated and nonlinear regression analysis was completed using GraphPad Prism software. C-H) CRC cells were transfected with miR-6883 under normoxia or hypoxia (<0.5% O _2_ ) and protein levels of CDK4/6, HIF1α, Glut1, and Ran were measured by Western blot.

## Description

Colorectal Cancer (CRC) is characterized by the proliferation of abnormal cells in the colon or rectum. CRC originates from small polyps, which are often benign but can become cancerous (American Cancer Society, 2020) . In 2023, there were an estimated 106,970 new cases of colon cancer and 46,050 new cases of rectal cancer in the United States, although recently incidence has been decreasing around 1% each year likely due to early screening, fecal immunochemical tests (FIT), and colonoscopies (American Cancer Society, 2023b; Roselló et al., 2019). Mortality is also decreasing; the number of CRC-related deaths in the United States in 2023 is estimated to be 52,550 compared to 53,200 in 2020 (American Cancer Society, 2020; Siegel et al., 2023) . However, CRC remains the third most diagnosed cancer and the second leading cause of cancer-related mortality in the United States (American Cancer Society, 2023b). Colorectal cancer incidence has been rising over the last two decades among younger individuals for unclear reasons (American Cancer Society, 2020) . Though treatments for CRC such as surgery, chemotherapy, targeted therapy, immunotherapy, and radiation can help to prolong patients’ lives, additional treatment options are needed for this deadly disease (Mayo Clinic, 2023) . Excluding skin cancers, breast cancer is the most common cancer in women (American Cancer Society, 2023a) and has a 5-year survival rate of 30% once it spreads to distant organs (American Cancer Society, 2023c). Breast cancer is normally treated with surgery, chemotherapy, hormone therapy, or radiation but new treatments are needed to improve survival outcomes while avoiding treatment-related toxicities (Mayo Clinic, 2022) .

Micro-RNAs (miRs) are a group of non-coding RNAs that can control the translation of coding genes by binding to mRNA and preventing its translation (Mehrgou et al., 2021) . miRs can bind oncogenes or tumor suppressors to modulate the initiation, progression, metastasis, and recurrence of CRC (To et al., 2018) . Current clinical trials are investigating the efficacy of therapeutic miRs in various types of cancer such as lymphoma, mycosis fungoides, melanoma, lung cancer, liver cancer, and myeloma (Menon et al., 2022; O'Neill, 2016; Zhang et al., 2021).

The tumor microenvironment often becomes hypoxic due to rapid oxygen consumption by tumors as well as the formation of atypical tumor blood vessels (Li et al., 2021; Muz et al., 2015) . When cancer cells detect a hypoxic environment, hypoxia-inducible factor 1 alpha (HIF1α) is stabilized (Cao et al., 2009) . Hypoxic conditions and HIF1α signaling have a pro-angiogenic effect and contribute to the formation of endothelial cells, which improves the delivery of oxygen and nutrients to the tumor (Krock et al., 2011) .

Cyclin-dependent kinases (CDKs) play a crucial role in cell cycle regulation. They bind cyclins, allowing transitions between G1, S, G2, and mitosis. Dysregulation of CDK activity contributes to the development of cancer. CDK4/6 inhibitors block cancer cell proliferation by inducing cell cycle arrest at G1 (Goel et al., 2018) . CDK4/6 is targeted by several FDA-approved cancer therapeutics including palbociclib, ribociclib, and abemaciclib. Previously, our lab has shown that CDK4/6 inhibition destabilizes HIF1α in CRC cells (Zhao et al., 2021) and that miR-6883 can inhibit CDK4/6 (Lulla et al., 2017) . We hypothesized that miR-6883 downregulates CDK4/6 and HIF1α in CRC and BC cells.

First, we confirmed that CRC cells are sensitive to CDK4/6 downregulation. HCT116, HT29, and SW480 cells were treated with increasing doses of the CDK4/6 inhibitor palbociclib for 72 hours and a CellTiter-Glo assay was used to measure cell viability. Bioluminescent imaging (panel A) and analysis in GraphPad Prism (panel B) revealed that CRC cells are sensitive to palbociclib (IC50 = ~8 µM), providing a rationale to test the CDK4/6-targeting miR-6883 as a potential therapeutic agent for this disease. As CDK4/6 inhibitors are already used to treat patients with BC, it was not necessary to confirm their sensitivity to palbociclib.

We evaluated the effect of miR-6883 on CDK4/6 levels in CRC (HT29, HCT116) and BC (MDA-MB-231) cells. We transfected the tumor cells with miR-6883 using lipofectamine RNAiMAX and measured protein levels of CDK4/6 and HIF1α by western blot. After testing different cell densities and transfection times, we selected transfection conditions that successfully downregulated CDK4/6. In HCT116 cells, miR-6883 downregulation of CDK4/6 (panel C lanes 1-2, panel F lanes 3-4, panel G lanes 3-4) co-occurred with downregulation of HIF1α (panel C lanes 1-2, panel G lanes 3-4) and HIF1α target gene Glut 1 (panel G lanes 3-4). Similar results were obtained with HT29 cells, in that miR-6883 downregulated CDK4/6 (panel D lanes 1-2) and HIF1α (panel E lanes 7-8). miR-6883 transfection was unsuccessful in SW480 CRC cells in the experiment shown (panel D lanes 9-10, panel F lanes 9-10). In MDA-MB-231 BC cells, transfection of miR-6883 caused downregulation of CDK4/6 which co-occurred with HIF1α downregulation (panel H lanes 11-12) with no effect on HIF1α target gene Glut1. Transfection of MCF7 (panel H, lanes 1-4) and MDA-MB-468 (panel H lanes 5-8) BC cells was unsuccessful in the experiments shown with no expected effect on HIF1α.

In summary, our results indicate that miR-6883 targets CDK4/6 in CRC (HCT116 and HT29) and BC (MDA-MB-231) cells. Downregulation of CDK4/6 co-occurred with downregulation of HIF1α, pointing to miR-6883 as a promising therapeutic for CRC and BC tumors with CDK4/6 and/or HIF1α hyperactivity.

## Methods

*Cell lines and culture conditions*

CRC (HT29, HCT116, and SW480) and BC (MCF7, MDA-MB-468 and MDA-MB-231) cells were obtained from ATCC. HT29 and HCT116 cells were grown in McCoy’s 5A Medium supplemented with 10% FBS and 1% penicillin/streptomycin. MCF7 cells were grown in Minimum Essential Eagle Medium MEM 1X supplemented with 10% FBS and 1% penicillin/streptomycin. SW480, MDA-MB-468 and MDA-MB-231 cells were grown in DMEM media supplemented with 10% FBS, 1% sodium pyruvate, and 1% penicillin/streptomycin. All cells were incubated at 37°C, 5% CO _2_ .

*CellTiterGlo Assay*

CRC cells were plated at a density of 5,000 cells per well of a 96-well plate. Cells were treated with palbociclib using doses ranging from 0-20 μM. Cell viability was measured by adding CellTiter-Glo reagent followed by bioluminescence imaging.

*Western Blot*

Cells were harvested and lysed using a RIPA buffer containing a protease inhibitor. Denaturing sample buffer was added, samples were boiled at 95°C for 10 minutes, and an equal amount of protein lysate was electrophoresed through 4-12% SDS-PAGE gels (Invitrogen) then transferred to PVDF membranes. The membrane was blocked with 5% milk in 1 × TTBS, incubated overnight in the appropriate primary antibody (HIF1α, pRb, Glut1, CDK4/6, or Ran), and incubated in the appropriate HRP-conjugated secondary antibody for two hours. The levels of antibody binding were detected using ECL western blotting detection reagent and the Syngene imaging system.

*Transfection*

CRC cells (HT29, HCT116 and SW480) and BC cells (MCF7, MDA-MB-468 and MDA-MB-231) were plated at various cell densities and incubated for at least 12 hours to allow cells to adhere to the plate. The cells were then transfected with 50 nmol/L miR-6883 mimic (Sigma-Aldrich HMI2616) using Lipofectamine® RNAiMAX Reagent. The cells were incubated for either 48 or 72 hours under normoxia (~20.9% O _2_ ) and hypoxia (<0.5% O _2_ ).

*miR-6883*

miR-6883 mimic was purchased from Sigma-Aldrich (HMI2616). This double-stranded RNA molecule mimics endogenous mature miRNA when introduced into cells.

## Reagents

**Table:** 

**Antibody**	**Source**	**Manufacturer and catalog #**
Anti-HIF1α	Mouse	Becton Dickinson 610958
Anti-Glut1	Rabbit	Cell Signaling 73015
Anti-CDK6	Mouse	Cell Signaling 3136
Anti-CDK4	Rabbit	Cell Signaling 12790
Anti-Ran	Mouse	Becton Dickinson 610341
Anti-mouse IgG	Goat	Thermo Fisher Scientific 31430
Anti-rabbit IgG	Rabbit	Thermo Fisher Scientific 31460
